# Using Medicare Data to Inform Intervention and Care Delivery for the Most Expensive Patients

**DOI:** 10.1093/geroni/igaa057.3350

**Published:** 2020-12-16

**Authors:** Sam Amodeo, Henrik Kowalkowski, Halley Brantley, Lauren Bangerter, Nicholas Jones, David Cook

**Affiliations:** 1 UnitedHealth Group, Eagan, Minnesota, United States; 2 UnitedHealth Group, Minneapolis, Minnesota, United States; 3 UnitedHealth Group, Minnetonka, Minnesota, United States; 4 Brown University School of Public Health, Providence, Rhode Island, United States; 5 United Health Group, Edina, Minnesota, United States

## Abstract

Older adults with high medical spend require tailored interventions and care delivery models to meet their complex needs. Segmenting high-spend patients is a promising approach to designing such interventions. In this study we explored patient spend across 4 years (2016-2019) using claims from 799,205 patients continuously enrolled in UnitedHealth Group Medicare Advantage (mean age=73.7; S.E.=0.01). Patients with healthcare spend in the top decile were segmented into three subgroups: catastrophic, persistent, and semi-persistent. Catastrophic patients had more acute events (acute myocardial infarction and hip/pelvic fracture) driving their cost. Persistent patients were younger (mean age=67.8; S.E.=0.06) and had significantly more medications. Semi-persistent patients were older (mean age=76.6; S.E.=0.04) and had significantly more chronic conditions and frailty, indicating their cost was driven by medical complexity. These subgroups displayed different temporal stability in their healthcare costs over time. Each year, 79-81% of the catastrophic group dropped out of the top decile. In contrast, nearly 72% of the persistent group remained in the top decile whereas only 37% of the semi-persistent group remained year over year. As the global population continues to age, it will be necessary to design interventions and care delivery models that address the complex needs of older adults in the high-spend patient population. Our study suggests that segmenting high-spend patients into potentially actionable subgroups is an important first step in achieving these goals.

